# Research on Target Deviation Measurement of Projectile Based on Shadow Imaging Method in Laser Screen Velocity Measuring System

**DOI:** 10.3390/s20020554

**Published:** 2020-01-19

**Authors:** Wenbo Chu, Donge Zhao, Baowei Liu, Bin Zhang, Zhiguo Gui

**Affiliations:** 1 Key Laboratory of Electronic Testing Technology for National Defense Science and Technology, North University of China, Taiyuan 030000, China; icwb0707@163.com (W.C.); zhangbinsmart@163.com (B.Z.); guizhiguo@nuc.edu.cn (Z.G.); 2 Experimental Testing Institute, China North Industries Group Corporation Limited, Weinan 714000, China; lbws2002@163.com

**Keywords:** shadow imaging, velocity measurement, beam collimation, sequential control, triggering, target deviation measurement

## Abstract

In the laser screen velocity measuring (LSVM) system, there is a deviation in the consistency of the optoelectronic response between the start light screen and the stop light screen. When the projectile passes through the light screen, the projectile’s over-target position, at which the timing pulse of the LSVM system is triggered, deviates from the actual position of the light screen (i.e., the target deviation). Therefore, it brings errors to the measurement of the projectile’s velocity, which has become a bottleneck, affecting the construction of a higher precision optoelectronic velocity measuring system. To solve this problem, this paper proposes a method based on high-speed shadow imaging to measure the projectile’s target deviation, ΔS, when the LSVM system triggers the timing pulse. The infrared pulse laser is collimated by the combination of the aspherical lens to form a parallel laser source that is used as the light source of the system. When the projectile passes through the light screen, the projectile’s over-target signal is processed by the specially designed trigger circuit. It uses the rising and falling edges of this signal to trigger the camera and pulsed laser source, respectively, to ensure that the projectile’s over-target image is adequately exposed. By capturing the images of the light screen of the LSVM system and the over-target projectile separately, this method of image edge detection was used to calculate the target deviation, and this value was used to correct the target distance of the LSVM to improve the accuracy of the measurement of the projectile’s velocity.

## 1. Introduction

Accurate measurement of a projectile’s velocity is an important part of the performance testing technology of weapons as it has a profound impact on the research, production, application, and development of weapons [1,2,3,4]. Therefore, research on the measuring method of the projectile’s velocity and how to improve the measuring accuracy of the projectile’s velocity have always been the key contents in the field of the weapon’s performance evaluation [5,6,7]. At present, the measuring methods of the projectile’s velocity are mainly to use interval velocity measuring devices [8,9,10,11]. The laser screen velocity measuring (LSVM) system is a typical interval velocity measuring device. It is the mainstream method of measuring a projectile’s velocity because of its real-time, non-contact, high precision [12,13,14]. The method needs to measure the time interval of the timing pulse generated by the projectile passing through the two light screens, and then calculate the projectile’s velocity, according to the distance between the two light screens (i.e., the target distance). However, when the projectile passes through two light screens. Due to the inconsistent optoelectronic response of the two light screens, the projectile’s over-target position, at which the timing pulse of the LSVM system is triggered, deviates from the actual position of the light screen (i.e., the target deviation), resulting in the distance that the projectile flies within the interval of the two timing pulses not being equal to the actual target distance. This introduces an error to the measuring accuracy of the projectile’s velocity, and has become a bottleneck problem for improving the measuring accuracy of the projectile’s velocity.

When constructing a high-accuracy optoelectronic velocity measuring system, the error caused by the target deviation cannot be ignored. At present, related research has only measured the response speed of the optoelectronic signal processing circuit of the two light screens of the optoelectronic velocity measuring system under laboratory conditions, and through the circuit design, the detected response speed of the two light screens can be made as consistent as possible. However, this type of research does not ensure that the projectile flies within the interval of the two timing pulses is equal to the actual target distance. In addition, during the research process, we found that due to the inconsistencies in the thickness uniformity of the two light screens in the emission path, the specific positions where the projectiles are triggered will also not be exactly the same. Considering the influence of wind speed and other environmental factors, the difference in the position of the projectile passing through the start light screen and the stop light screen will also become larger. Therefore, the current research on the optoelectronic response speed on the circuit cannot ensure that the optoelectronic response of the projectile passes through the two light screens is consistent, which makes the current research unable to ensure the improvement of the accuracy of the velocity measurement, and the measurement of target deviation must be combined with the comprehensive analysis at the measuring site of the system. Moreover, the projectile’ velocity is fast, and it is difficult to accurately measure the actual fly distance within the time interval between two pulse signals. The light screen of the virtual entity that the projectile passes through causes the system to generate the trigger signal, and the light screens of these two virtual entities are difficult to image, which brings greater test difficulty for the measurement of the target deviation.

In order to measure the target deviation, the research in this paper used the method of shadow imaging in the measurement. At present, shadow imaging is often used in real-time imaging detection. It is an imaging method that uses the optical principle of light traveling in a straight line to obtain the projection of the sample through the optical detector to characterize its physical form. In the use of shadow imaging to capture high-speed objects, some researchers have used the femtosecond laser to illuminate the side of the flying path of the flyer, capture it with the Intensified Charge Coupled Device (ICCD) on the other side, and the delay generator is used to control the time to obtain the relatively ideal side image of the flyer at the corresponding time during the high-speed motion [15]. Relevant researchers have also used a parallel laser with the wavelength of 589 nm to match the size of the flow field, where it was imaged on a high-speed camera by the imaging lens. The camera was triggered in manual mode, and was operated to enter the recording state with a long exposure before the experiment to capture the situation of an explosive explosion in the air environment with a delay of 100 ms [16]. In addition, there have also been relevant studies conducted to measure the debris cloud at high-speed collisions by using wait-type imaging, where the camera was turned on to expose it before the experiment. When the projectile hit the target, the laser source flashed and the shape of the debris cloud generated by the impact was recorded on the Charge Coupled Device (CCD). The flash signal of the laser light source is provided by the detector for detecting projectiles, and the time signal of the moment when the projectile hits the target is provided by the optical radiation detector [17].

This paper was based on the research into the parallel illumination of a high-power infrared narrow-pulse laser, the signal trigger, the sequential control, and the reception of the image sensor. We proposed a method based on high-speed shadow imaging to obtain the image of the projectile’s position when the LSVM system generates the timing signal [18,19,20,21,22], which uses the image processing to obtain the target deviation of the projectile relative to the light screen, thereby correcting the target distance of the LSVM system. It is intended to measure and calibrate the consistency of the optoelectronic response of the LSVM system by the proposed method, and improve the measuring accuracy of the projectile’s velocity.

## 2. Principle

**Figure 1:**
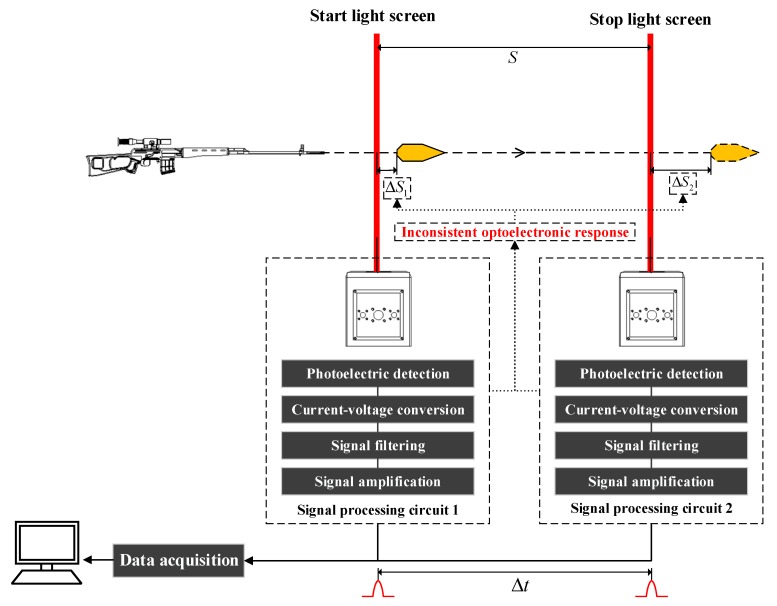
The principle and target deviation diagram of the laser screen velocity measuring (LSVM) system.

The LSVM system adopts the optoelectronic non-contact interval velocity measuring method. As shown in Figure 1, both measuring hosts use the laser as the light source to generate two mutually parallel light screens (start light screen and stop light screen). When the projectile flies through the two light screens, it blocks part of the light, respectively, the changing light flux is converted into the electrical signal by the photoelectric sensor of the measuring host, and the signal of the projectile passing the light screen is formed by the signal processing circuit; the corresponding algorithm is used to calculate the time interval Δ*t* between the two light screens when the projectile flies through the two light screens, based on the distance *S* between the two light screens, which is measured with a steel ruler or a laser interferometer (i.e., target distance); and the average velocity of the projectile passing through the two light screens is obtained from v=S/Δt. However, due to the deviation in the consistency of the optoelectronic response between the start light screen and the stop light screen, when the projectile passes through the light screen, the projectile’s over-target position when the timing pulse is triggered deviates from the actual position of the light screen, resulting in the distance that the projectile flies within the interval of the two timing pulse not being equal to the actual target distance, where the deviation is $\Delta S_{2}-\Delta S_{1}$.

**Figure 2:**
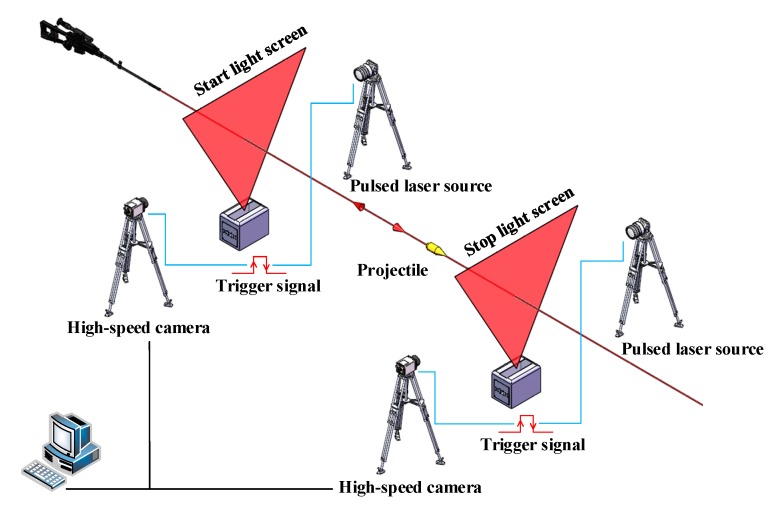
Measuring site of target deviation.

In order to reduce the influence of the target deviation on the measuring accuracy of the projectile’s velocity, the target deviations of the start light screen and stop light screen are obtained using the shadow imaging method to obtain the projectile’s over-target image when the LSVM system generates the timing signal, and by image processing. Its measuring site is shown in Figure 2, Using the high-speed camera as the measuring core, increasing the sensitivity of the camera with the pulsed laser source allows the camera to capture a clear image of a high-speed projectile with extremely short exposure times. The output signal by the signal processing circuit of the LSVM system is used as the trigger signal of the camera and the pulsed laser source [23], and the two can cooperate with each other in timing to accurately acquire the projectile’s over-target image at the timing moment.

**Figure 3:**
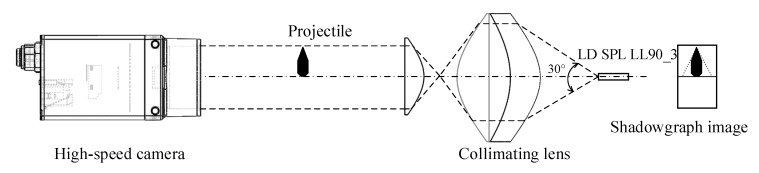
Schematic diagram of shadow imaging.

When the system is working, as shown in Figure 3, after the pulsed laser source is collimated by the optical system, a high-precision parallel laser source is formed, and the entire image sensor is covered by the parallel laser, and a bright area is formed on the image sensor. When the projectile passes through the image surface, part of the parallel light is blocked, and a dark area forms on the image sensor. The boundary between the bright and dark areas is the projection of the projectile [24,25,26,27,28,29]. The position of the projection of the light screen and the position of the projectile on the image sensor are separately recorded by the high-speed camera [30,31,32], and the target deviation of the projectile can be obtained according to the pixel value of the image sensor.

## 3. Key Device Analysis

### 3.1. Imaging Module

To achieve high-resolution imaging of the high-speed projectile, a high-speed camera is a vital component. This measuring method is to use the high-speed camera for the POINT GREY series GS3-U3-23S6. The main parameters are as follows: the image sensor is IMX-174, the pixel size is about 5.86 μm × 5.86 μm, the resolution is 1920 × 1200, the shortest exposure time is 4 μs, the sensitivity of the image sensor is 13 μV/e^−^, and the working mode is free running and external triggering.

### 3.2. Laser Source Module

(1) Pulsed laser source

The pulsed semiconductor laser is responsible for providing auxiliary illumination in this measuring system to ensure that the camera is provided with the energy needed for ideal imaging with an extremely short exposure time. In order to prevent the occurrence of smear ghost when capturing the image of the high-speed projectile, the pulse width of the pulse laser is required to be as narrow as possible, while ensuring sufficient energy. If the projectile’s velocity is *V* and the pulse duration of the laser is *T*, the displacement of the projectile during the exposure time is *X*, when the distance of the projectile moving in the image is not more than the length of a pixel of the image sensor, the smear ghost can be ignored. According to Equation (1), a is the length of a pixel of the image sensor, if the projectile’s velocity is *V* = 1000 m/s, the pulse duration of the laser is *T* = 6 ns.

(1) T=a/2V

Therefore, in order to reduce the influence of visible light on imaging, combined with the spectral response characteristics of the image sensor, this system uses an OSRAM’s SPL LL90_3 pulsed semiconductor laser. The main parameters are as follows: the wavelength is 905 nm, the meridian divergence angle is $\theta_{m}=30^{o}$, the sagittal divergence angle is $\theta_{s}=15^{o}$, and the peak power is 70 W. These were matched with the driving circuit of the pulsed laser source to realize an optical pulse with a pulse width of 6 ns.

(2) Beam collimation

According to the working principle of the semiconductor laser and the characteristics of the far-field beam, the laser beam spreads out at different angles in the meridian direction and the sagittal direction, resulting in the formation of an elliptical spot at the far-field. The beam quality is very poor as the positions of the beam waist of the semiconductor laser in two directions do not coincide, causing inherent dispersion. Therefore, this paper proposes a scheme for achieving collimation of the laser beam by using the combination of the aspherical lens.

**Figure 4:**
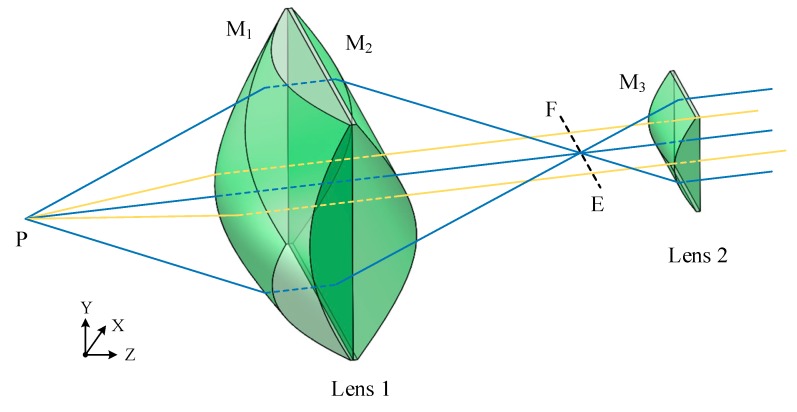
Design idea of laser source collimation.

This scheme uses two aspherical lenses to collimate the laser beam, the design idea is shown in Figure 4. In the lens combination, the front surface M_1_ of the first lens is a non-rotationally symmetrical curved surface, and is mainly responsible for forming a diverging laser beam into a parallel beam and eliminating the inherent dispersion. Its rear surface M_2_ is a hyperbolic cylindrical surface, which mainly compresses the light in the meridian direction without changing the light in the sagittal direction. The light formed after shaping through the first lens intersects the optical axis and forms a line source EF. The second lens is a hyperbolic cylindrical lens, which mainly realizes light collimation in the meridian direction without changing the light in the sagittal direction.

**Figure 5:**
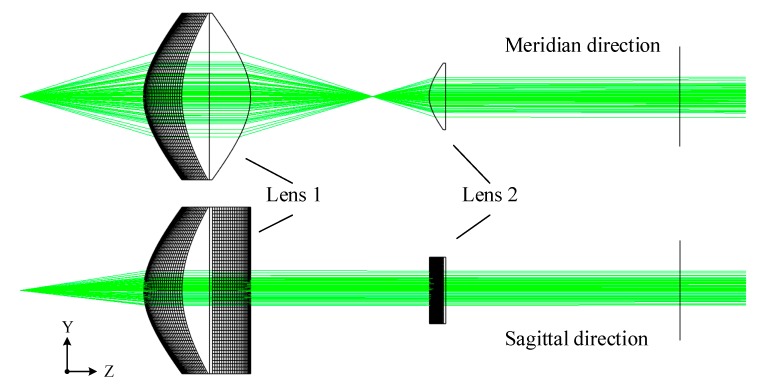
Zemax simulation of the collimated light source.

**Figure 6:**
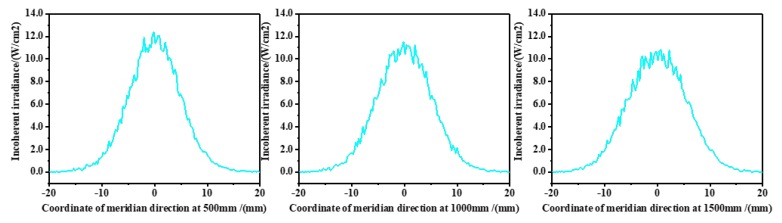
Distribution curves of light intensity in the meridian direction (500 mm, 1000 mm, and 1500 mm from the collimation system in the Z-axis direction).

**Figure 7:**
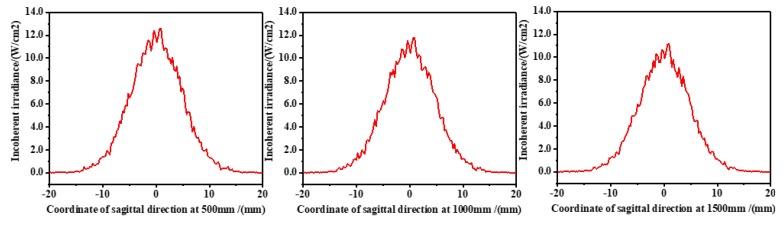
Distribution curves of light intensity in the sagittal direction (500 mm, 1000 mm, and 1500 mm from the collimation system in the Z-axis direction).

According to the design idea, the mathematical models were established for the meridian direction and the sagittal direction of the lens, and Fermat’s theorem and the aspherical equation were used to solve the curve equations of each face of the combination of the collimating lens. The specific mathematical derivation is not described here. The Zemax simulation is shown in Figure 5. This detected the distribution of the light intensity at positions of 500 mm, 1000 mm, and 1500 mm from the collimation system in the Z-axis direction. The curves of the results are shown in Figure 6 and Figure 7.

The distribution of the light intensity is the Gaussian distribution, and $1/e^{2}$ of the peak of light intensity was selected as the boundary of the spot radius. According to the curves, in the meridian direction, the spot radius at 500 mm was 9.64 mm, and the spot radius at 1500 mm was 10.36 mm, and in the sagittal direction, the spot radius at 500 mm was 9.40 mm, and the spot radius at 1500 mm was 9.72 mm. Through calculation, the collimation system of the laser source had a half divergence angle of 0.72 mrad in the meridional direction and a half divergence angle of 0.32 mrad in the sagittal direction, so the effect of collimation was good.

### 3.3. Trigger Module

(1) Trigger circuit

**Figure 8:**
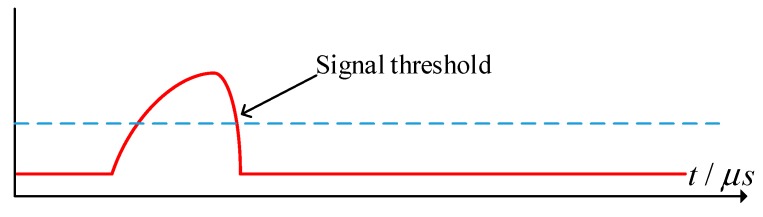
Projectile’s over-target analog signal.

**Figure 9:**
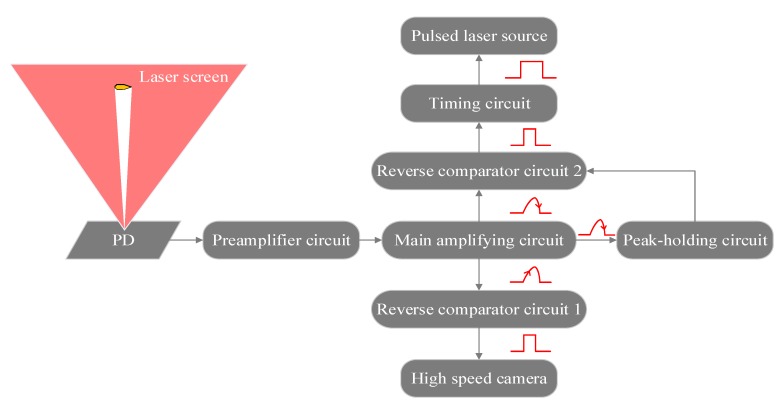
Relationship between circuits in the trigger module.

In order to capture the high-speed projectile’s over-target image, a sharp TTL signal level is needed as the trigger source for the camera and the pulsed laser source. In this system, the trigger circuit is shown in Figure 8. When the projectile passes through the light screen, part of the light is blocked, and the light intensity received by the photodetector changes to produce a weak electrical signal. This electrical signal is converted into a voltage signal by the preamplifier circuit and the main amplifying circuit; after noise reduction and amplification, the analog signal of the projectile’s over-target is formed, as shown in Figure 8. The relationship between the circuits in the trigger module is shown in Figure 9.

The signal threshold is set in the reverse comparison circuit 1, and the principle of threshold setting is adjusted according to the amplitude of the signal noise. When the value of the input voltage is lower than the threshold, the low level is the output. Conversely, when the value of the input voltage is higher than the threshold, the high level is the output. Through this circuit, the analog signal of the projectile’s over-target is converted into a digital pulse signal that triggers the camera.

In the measuring system, the time node of 1/2 of the falling edge of the analog signal of the projectile’s over-target was used as the timing moment of the velocity measurement. In order to capture the projectile image at this moment, it is necessary to trigger the pulsed laser source at this moment, and the reverse comparison circuit 2 cooperates with the peak hold circuit to give the time node of 1/2 of the falling edge of the analog signal of the projectile’s over-target, and a high-level pulse signal is generated through the timing circuit to satisfy the trigger condition of the pulsed laser source.

(2) Sequential control

**Figure 10:**
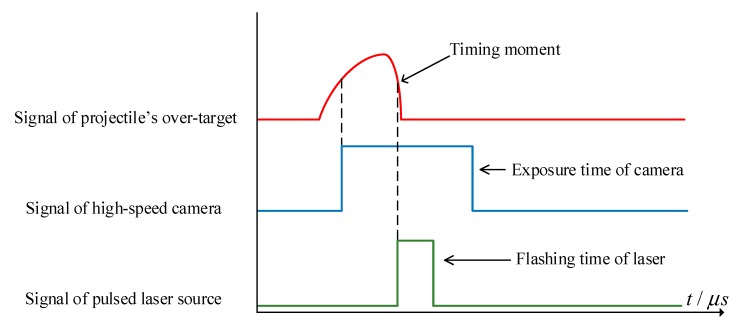
Schematic diagram of sequential control.

In order to prevent the camera shutter not turning on when the pulsed laser source is illuminated, the exposure of the camera is insufficient to capture the clear projectile’s over-target image. This measuring scheme uses asynchronous sequential control to trigger the pulsed laser source when the camera shutter is opened. The sequential control is shown in Figure 10. When the projectile passes through the light screen, according to the trigger circuit introduced in the previous section, the LVCM system generates the projectile’s over-target signal. A trigger pulse of the camera is generated at the rising edge of the projectile’s over-target signal, and it is controlled to open the shutter of the camera. When the amplitude of the projectile’s over-target signal drops to 1/2 of the peak value, the trigger pulse of the laser source is generated (the time node that generates this pulse can also be used as the timing moment for calculating the velocity of the projectile in the LSVM system). It controls the pulsed laser source to illuminate, and cooperate with the camera to capture the clear projectile’s over-target image.

## 4. Sensor Receiving Energy Analysis

In this measuring scheme, the laser source is composed of a pulse laser and collimating lens combination, and is responsible for filling light for the camera during imaging. It is necessary to calculate the energy that the laser source projects onto the camera image sensor, and whether it can capture the clear image of the projectile’s over-target in a very short exposure time.

After optical shaping of the SPL LL90_3 pulsed semiconductor laser, the total power of the pulse laser that reaches the image sensor after air transmission is:

(2) $$p_{1}=p_{0}\eta_{1}t_{1}t_{2}t_{3}$$

The peak power of SPL LL90_3 is $p_{0}=70\;W$. The emission efficiency of the laser is $\eta_{1}=0.9$, the transmittance of the optical system is $t_{1}=0.85$, the coefficient after air transmission loss is $t_{2}=0.9$, the image receiving ratio is $t_{3}=0.42$, and then, according to Equation (2), the image receiving light power is $P_{1}\approx 20\;W$.

The IMX-174 image sensor has a pixel size of 5.86 μm × 5.86 μm, the number of pixels of the image sensor is H=1920×1200, and the incident power per pixel is:

(3) $$p_{2}=\frac{p_{1}}{H}=\frac{20}{1920\times 1200}=8.68\;\mu W$$

The wavelength of SPL LL90_3 is λ=905 nm, and so the frequency of the laser is:

(4) $$f=\frac{c}{\lambda }=\frac{3\times 10^{8}m/s}{905\;\mathrm{nm}}=3.313\times 10^{14}\;\mathrm{Hz}$$

According to the micro energy formula p=h×f×n, the number of photons that can be generated on a single pixel of the image sensor is:

(5) $$n=\frac{p_{2}}{h\times f}=\frac{8.68\times 10^{-6}}{6.62\times 10^{-34}\times 3.313\times 10^{14}}=3.958\times 10^{13}\;e^{-}$$

where h is the Planck constant; f is the frequency of the laser; and n is the number of photons.

According to the quantum efficiency curve of the image sensor IMX-174, it is known that the quantum efficiency is about $\eta_{2}=0.07$ at the wavelength of 905 nm, the pulse width of SPL LL90_3 is T=6 ns, the sensitivity of the image sensor is $k=13{\;\mathrm{uV}/e}^{-}$, and then in 6 ns, the output voltage of a single pixel is:

(6) $$V=n\times \eta_{2}\times k\times T=0.22\;V$$

According to the manual of the IMX-174 image sensor, the range of the output voltage of a single pixel is 0.57 mV–0.6 V. The laser source used in this measuring scheme has an output voltage of 0.22 V on a single pixel in 6 ns, which satisfies the energy demand and can be ideally imaged.

## 5. Experiment and Results

### 5.1. Experiment

**Figure 11:**
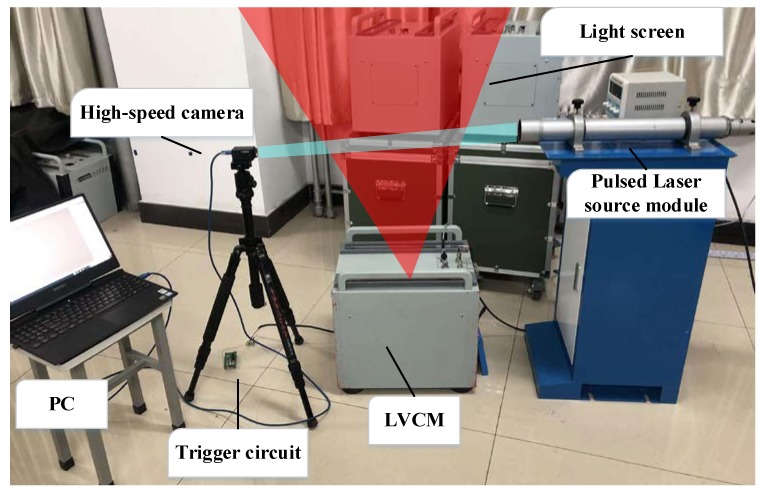
Experimental layout.

According to the above analysis, the measuring scheme is mainly composed of a laser source module, high-speed camera, trigger circuit and PC, wherein the laser source module is composed of a high-power narrow-pulse semiconductor laser and collimation system. The experimental components and experimental layout are shown in Figure 11.

**Figure 12:**
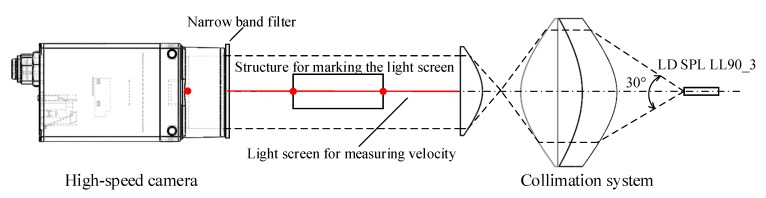
Method of coaxial adjustment.

In order to adjust the image sensor, laser source module, and light screen so that they are coaxial, a special structure for marking the light screen is placed at the position of the ballistic line in the light screen (the structure is engraved with the 1 mm aperture slit on both sides, which is responsible for calibrating the position of the light screen). As shown in Figure 12, when the parallel laser source illuminates the structure, the apertures on both sides are projected onto the image sensor and form a bright area. When the projections of the apertures on both sides coincide, the laser source module, the light screen, and the image sensor are adjusted to be coaxial, and the position of the light screen is calibrated. It can also be imaged by the image sensor and determines the actual position of the light screen, which is the virtual entity.

**Figure 13:**
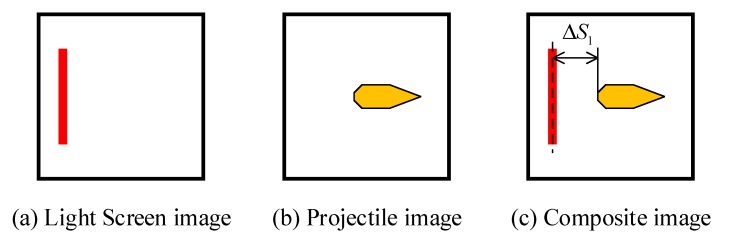
Acquisition process of target deviation.

The image of the position of the light screen is photographed statically (as shown in Figure 13a), then the structure for marking the light screen is removed, and the image of the projectile’s over-target is captured under dynamic conditions (as shown in Figure 13b). The two images were synthesized to obtain the image as shown in Figure 13c. The measured value of the target deviation is:

(7) ΔS=lN

where l is the size of a single pixel in the image sensor, and N is the number of pixels between the center of the light screen and the projectile’s tail. 

Through the image processing, the target deviation $\Delta S_{1}$ of the start light screen and the target deviation $\Delta S_{2}$ of the stop light screen can be obtained. By shooting a set of projectiles, the average of the difference in the target deviations between the start light screen and the stop light screen is calculated as $\bar{\Delta S_{1}-\Delta S_{2}}$, and the corrected target distance is $S-\bar{\left(\Delta S_{1}-\Delta S_{2}\right)}$.

### 5.2. Image Processing

(1) Image denoising

Random noise is generated when shooting the projectile, and there is image noise caused by its own dark current during image acquisition. For the noise existing in the measuring system, the image is preprocessed by a low-pass linear mean filter to eliminate noise. The equation is:

(8) $$f^{\prime }(x,y)=\frac{1}{9}\sum_{i=-1}^{1}f(x+i,y+j)$$

This uses a 3 × 3 template to scan the image, and replaces the gray value of the pixel at the center of the template with the average to eliminate the noise of the image.

(2) The tail coordinate extraction

The Sobel operator is used to obtain the edge of the projectile’s tail. The Sobel operator consists of a horizontal operator and a vertical operator, which are allowed to do the plane convolution with the image separately, which can get the difference approximation of the horizontal and vertical brightness. In Equations (9) and (10), A represents the original image, and *Gx* and *Gy* represent the gray value of the image after the horizontal and vertical edge detection, respectively.

(9) $$G_{x}=\left[\begin{array}{ccc} -1 & 0 & +1\\ -2 & 0 & +2\\ -1 & 0 & +1 \end{array}\right]*A$$

(10) $$G_{y}=\left[\begin{array}{ccc} +1 & +2 & +1\\ 0 & 0 & 0\\ -1 & -2 & -1 \end{array}\right]*A$$

The template of the neighborhood of the projectile’s image is as shown in Equation (11).

(11) $$\left[\begin{array}{ccc} f(x-1,y-1) & f(x-1,y) & f(x-1,y+1)\\ f(x,y-1) & f(x,y) & f(x,y+1)\\ f(x+1,y-1) & f(x+1,y) & f(x+1,y+1) \end{array}\right]$$

The operation of the horizontal convolution is:

(12) $$\begin{array}{ll} \Delta G_{x} & =\left[f(x-1,y+1)+2f(x,y+1)+f(x+1,y+1)\right]\\ & -\left[f(x-1,y-1)+2f(x,y-1)+f(x+1,y-1)\right] \end{array}$$

The operation of the vertical convolution is:

(13) $$\begin{array}{ll} \Delta G_{y} & =\left[f(x+1,y-1)+2f(x+1,y)+f(x+1,y+1)\right]\\ & -\left[f(x-1,y-1)+2f(x-1,y)+f(x-1,y+1)\right] \end{array}$$

The result of the gradient calculation is:

(14) $$G\left[f(x,y)\right]=\sqrt{\Delta G_{x}^{2}+\Delta G_{y}^{2}}$$

Using the calculative result, the edge information of the projectile is extracted, and the coordinate of the position of the projectile’s tail is obtained.

(3) Extraction of the center coordinates of the light screen

The image of the light screen is binarized and the threshold is obtained. The light screen is then segmented from the background by segmentation of the threshold. The center of the light screen is determined by solving its centroid. In order to calculate the distance of the light screen relative to the projectile’s tail in the direction of the abscissa, it is only necessary to determine the abscissa of the center of the light screen, and the following equation can be obtained:

(15) $$x=\frac{\sum_{i-1}^{n}p_{i}x_{i}}{\sum_{i-1}^{n}p_{i}}$$

where $x_{i}$ is the abscissa of each pixel of the light screen, the corresponding gray value is $p_{i}$, and x is the abscissa of the center of the light screen.

### 5.3. Error Analysis

**Figure 14:**
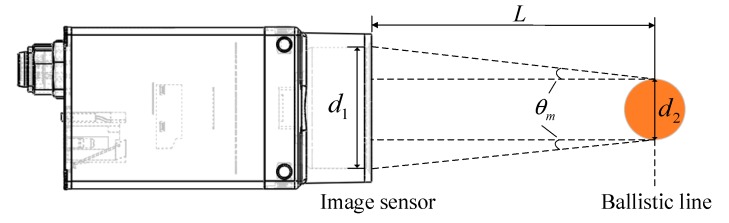
Error analysis.

The systematic error is mainly introduced by the non-parallelism of the laser source. As shown in Figure 14, the position of the ballistic line is *L =* 500 mm from the image sensor of the camera. Select the maximum divergence angle of the parallel laser source to be substituted into the error analysis, that is, the half divergence angle in the meridian direction is $\theta_{m}=0.72\;\mathrm{mrad}$. According to the equation:

(16) $$d_{2}=d_{1}-2Ltan\theta_{m}$$

The width of the image sensor is $d_{1}=11\;\mathrm{mm}$, the width that mapped to the position of the ballistic line is $d_{2}=10.28\;\mathrm{mm}$, and when the ballistic line position is mapped to the image sensor, each pixel represents the size $l=d_{2}/1920=5.35\;\mu m$.

### 5.4. Results

**Figure 15:**
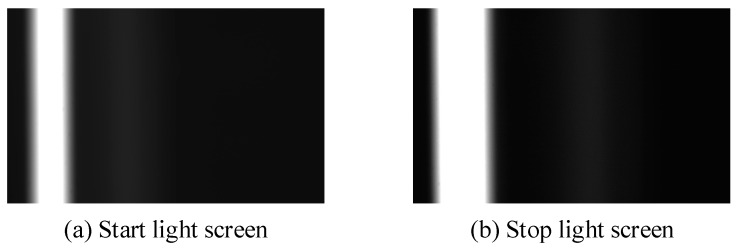
The images of the start light screen and stop light screen.

**Figure 16:**
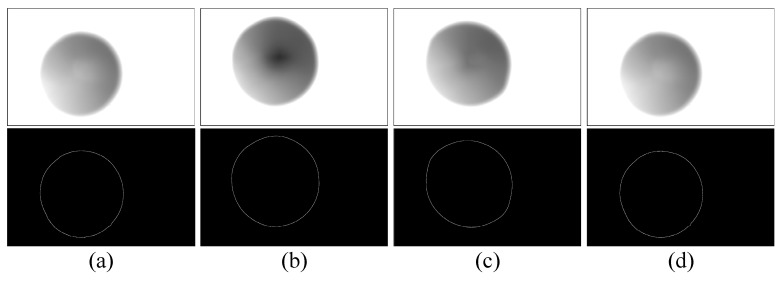
Projectile’s over-target image in the start light screen (**a**–**d**) are the projectile’s over-target images in the start light screen after firing four projectiles.

**Figure 17:**
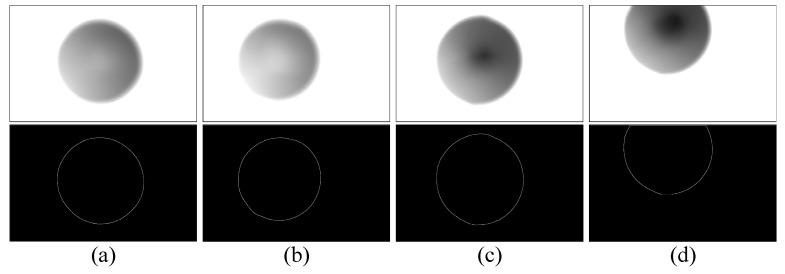
Projectile’s over-target image in the stop light screen (**a**–**d**) are the projectile’s over-target images in the stop light screen after firing four projectiles.

The images of the light screen taken with the aid of the structure for marking the light screen are shown in Figure 15. The 5.8 mm simulated projectile was used to shoot at the position of the ballistic line. The projectile’s over-target image taken at the position of the start light screen is shown in Figure 16, and the projectile’s over-target image taken at the position of the stop light screen is shown in Figure 17.

After image processing was performed (Figure 15, Figure 16 and Figure 17), the coordinates of the light screen and the projectile’s tail were obtained, as shown in Table 1 and Table 2. After calculation, the average target deviation of the start light screen was 0.33 mm, and the average target deviation of the stop light screen was 0.565 mm. It is known that the target distance between two light screens is 220 mm, after correction, the target distance of the LSVM system was 219.765 mm.

## 6. Conclusions and Discussion

In the optoelectronic system that uses multiple light screens to form an interval velocity measurement, there is a difference in the consistency of the optoelectronic response between the start light screen and the stop light screen because the optoelectronic response speeds of the photodetection circuits of the two light screens are different, and the two light screens have different thickness uniformities in the emission path. Therefore, when the projectile passes through the light screen, the projectile’s over-target position at which the timing pulse is triggered deviates from the actual position of the light screen. Therefore, error in the target distance in the velocity measuring system is introduced. 

In order to solve this problem, this paper first proposed the aspherical lens combination as the collimation of the laser source of the system. The light beam of the two dimensions are respectively collimated by different hyperboloids of the lens, which achieves the effect of eliminating dispersion and forms a parallel pulsed laser source with good collimation. Then, this paper designed the trigger circuit using asynchronous sequential control using the rising and falling edges of the projectile’s over-target signal in the LSVM system as the trigger signals for the high-speed camera and the pulsed laser source, respectively, to ensure that clear exposure images of high-speed projectiles are captured. A special structure should be designed for marking the light screen to calibrate the position of the light screen, which is the virtual entity, so that it can be imaged by the image sensor and determines the actual position of the light screen. Finally, this paper used the shadow imaging method to obtain the image of the projectile’s over-target at the timing moment, and calculated the target deviation of the start light screen and the stop light screen by image processing.

Through the proposed method, it re-corrects the actual target distance of the LSVM system (that is, the actual flight distance of the projectile within the timing pulse interval), eliminating errors caused by the inconsistent response of the optoelectronic detection of each light screen, thereby improving the measurement accuracy of the projectile’s velocity in the LSVM system. To our knowledge, in the field of projectile velocity measurement, this is the first time that the error due to the inconsistency in photoelectric response of the optoelectronic velocity measuring system has been analyzed and measured.

Compared with capturing the projectile’s over-target image by direct imaging with a pulsed light source, this method has the following advantages: low single-pulse energy of the light source, less error factors introduced on the target deviation measurement, easy traceability, and the image quality is independent of the material and posture of the projectile. At present, this method provides a way of thinking for the calibration measurement of the photoelectric velocity measuring system in the weapon measurement field. For large-caliber projectiles (with the caliber of the 12.7 mm or more), it is necessary to take a large-format and high-speed camera to capture a complete projectile’s over-target image, but due to the high cost of large-format cameras, the corresponding lens group can be matched in front of the small-format and high-speed camera. The parallel light of the large diameter is formed into the parallel light of the small diameter and is projected to the image sensor of the camera, but the calibration of the pixel size in the image sensor is required before the measurement.

## Figures and Tables

**Table 1:** Target deviation of start light screen.

No.	Projectile’s Tail Coordinates	Light Screen Coordinates	Pixel Size (μm)	Target Deviation (mm)	Average (mm)
a	332	264	5.35	0.36	0.33
b	316			0.28	
c	326			0.33	
d	330			0.35	

**Table 2:** Target deviation of stop light screen.

No.	Projectile’s Tail Coordinates	Light Screen Coordinates	Pixel Size (μm)	Target Deviation (mm)	Average (mm)
a	487	297	5.35	1.02	0.565
b	360			0.34	
c	412			0.62	
d	350			0.28	
